# Cerebral Venous Drainage in Patients With Space-Occupying Middle Cerebral Artery Infarction: Effects on Functional Outcome After Hemicraniectomy

**DOI:** 10.3389/fneur.2018.00876

**Published:** 2018-10-17

**Authors:** Volker Puetz, Johannes C. Gerber, Philipp Krüger, Matthias Kuhn, Heinz Reichmann, Hauke Schneider

**Affiliations:** ^1^Department of Neurology, University Hospital Carl Gustav Carus, Technische Universität Dresden, Dresden, Germany; ^2^Department of Anesthesiology, Klinikum Dortmund gGmbH, Dortmund, Germany; ^3^Carl Gustav Carus Faculty of Medicine, Institute for Medical Informatics and Biometry, Technische Universität Dresden, Dresden, Germany; ^4^Department of Neurology, Klinikum Augsburg, Augsburg, Germany

**Keywords:** stroke, middle cerebral artery infarction, space-occupying infarction, decompressive surgery, hemicraniectomy, cerebral venous drainage, cerebral sinus, cerebral veins

## Abstract

**Background:** Cerebral venous drainage might influence brain edema characteristics and functional outcome of patients with severe ischemic stroke. The purpose of the study was to evaluate whether hypoplasia of transverse sinuses or the internal jugular veins is associated with poor functional outcome in patients with space-occupying middle cerebral artery (MCA) infarction who underwent decompressive surgery.

**Methods:** We performed a retrospective analysis of patients with space-occupying MCA infarction treated with decompressive surgery at our university hospital. The transverse sinuses and the internal jugular veins were evaluated on baseline images and categorized as normal, hypoplastic or occluded. We defined composite variables for ipsilateral, contralateral or any abnormal cerebral venous drainage. We assessed the functional outcome at 12 months with the modified Rankin scale (mRS) score and defined poor functional outcome as mRS scores 5 and 6.

**Results:** We analyzed 88 patients with available baseline imaging data [mean [SD] patient age 53 (±9) years; median[IQR] time to decompressive surgery 31(22-51) h]. At 12 months 44 patients (50%) had a poor outcome. In univariate analysis neither ipsilateral (OR 1.98;95%CI: 0.75–5.40), nor contralateral (OR 1.56;95%CI: 0.59–4.24) or any (OR 1.6; 95%CI: 0.68–3.79) hypoplasia or occlusion of venous drainage were significantly associated with poor functional outcome. In multivariate analyses, higher patient age (OR 1.07;95%CI 1.01–1.14) and baseline stroke severity (OR 3.42;95%CI 1.31–9.40) were independent predictors of poor functional outcome, but not ipsilateral hypoplasia or occlusion of venous drainage (OR 1.31;95%CI 0.47–3.67).

**Conclusions:** The cerebral venous drainage pattern was not significantly associated with poor functional outcome in our cohort of patients with space-occupying MCA infarction who underwent decompressive surgery.

## Introduction

Space-occupying MCA infarction occurs in up to 10% of patients with supratentorial ischemic stroke (1). Brain edema formation of ischemic tissue exerts a mass effect with impending cerebral herniation and early clinical deterioration (2). Early decompressive surgery improves survival rates and functional outcome of patients with large MCA infarction (3–7). Conservative treatment strategies, e.g., osmotherapy, barbiturate sedation, or hypothermia, are less effective or without proven clinical benefit, respectively (8–10).

Space-occupying brain edema usually arises within 2 to 5 days after stroke onset, but the extent of brain edema varies (8). Previously, an abnormal ipsilateral cerebral venous drainage (CVD) has been shown to be associated with the development of fatal brain edema after internal carotid artery (ICA) or proximal MCA occlusion in a case series including 14 patients (11). In this series, 4 of 5 patients with a malignant edema had an abnormal ipsilateral CVD characterized by hypoplasia or occlusion of the transverse sinus or the internal jugular vein, whereas all 9 patients without malignant edema had normal ipsilateral CVD.

Factors affecting the clinical course and functional outcome after early decompressive surgery are not well understood. Prospectively acquired data on the effect of timing of surgery on clinical outcome are inconclusive (8). The impact of CVD on the development of infarct edema and the clinical course after decompressive surgery for space-occupying MCA infarction is unclear.

The aim of our study was to analyze the association of cerebral venous drainage patterns with the clinical course and functional outcome of patients with space-occupying MCA infarction after decompressive surgery. Our main hypothesis was that impaired venous drainage ipsilateral to the side of the infarction would result in edema expansion and subsequent poor functional outcome despite decompressive surgery.

## Methods

### Study design and patient selection

We performed a retrospective, single-center cohort study and used a local bone storage database to identify stroke patients aged 18 years and older with space-occupying MCA infarction who underwent decompressive surgery at our university hospital between 2001 and 2010. All patients with available baseline imaging datasets prior to hemicraniectomy [cerebral non-contrast CT (NCCT), CT angiography (CTA), magnetic resonance imaging (MRI), MRI angiography (MRA) and/or digital subtraction angiography (DSA)] were included into the analysis. Patients with missing clinical follow-up data were excluded.

### Stroke treatment and critical care

Patients were monitored and treated according to current European guidelines for the management of acute ischemic stroke and according to our institutional standard for treatment of space-occupying MCA infarction as follows: Decompressive surgery and ipsilateral intracranial pressure probe insertion was performed if patients had clinical signs of large MCA infarction, showed impaired consciousness, were 18 years or older, and if imaging revealed an infarction of >50% of the MCA territory. Postoperatively, all patients were treated at our neurocritical care unit. Therapeutic hypothermia was performed with a target core temperature of 33–34°C for 96 h if no contraindications were present. Further critical care measures were reported elsewhere (10). Briefly, sustained elevated intracranial pressure (>15 mmHg) and cerebral perfusion pressure below 70 mmHg were treated with osmotherapy (mainly mannitol 20% as short infusion every 4 to 6 h) and/or with vasopressors, respectively.

### Data retrieval and management

Baseline parameters and treatment measures were retrieved from our electronic patient management systems (ICM®, Dräger, Germany; ORBIS®, AGFA Healthcare, Germany) or paper patient files. Functional outcome at 12 months was evaluated retrospectively using structured telephone interviews and mailed questionnaires including the structured assessment of the modified Rankin scale sore. Discharge letters of rehabilitation centers and entries of registers of residents were used to verify outcome data including date of death.

### Image analyses

Analyses of imaging data was performed as a three reader consensus by two stroke neurologists (V.P., H.S.) and one neuroradiologist (J.C.G.), blinded for clinical and outcome data of individual patients. We evaluated the cerebral venous drainage via the transverse sinuses and the internal jugular veins in regard to the side of the MCA infarction and classified it into (1) occlusion (or aplasia), (2) hypoplasia, (3) normal appearance, and (4) non-availability due to technical reasons (e.g., CT scan level). Composite variables were defined for venous drainage: (A) *ipsilateral*, (B) *contralateral*, or (C) *any* occlusion/hypoplasia of internal jugular veins or transverse sinuses. Images acquired before hemicraniectomy were analyzed for the extent of MCA infarction using the Alberta Stroke Program Early CT Score (ASPECTS) and for the presence of additional infarction in vascular territories not represented by ASPECTS (12, 13). Postoperative images were analyzed for the size of hemicraniectomy (maximum outer diameter on NCCT). Imaging modalities were cerebral NCCT, CTA, MRI, MRA, and/or DSA, depending on availability. In patients where only NCCT or MRI was available, we diagnosed hypoplasia or occlusion of transverse sinus if the associated bony indentation was layed out less than 50% compared to the opposite side.

### Statistical analyses

Clinical and neuroimaging characteristics were compared between patients with poor long-term functional outcome (mRS 5–6 at 12 months) and patients with mRS 0–4 at 12 months. For group-comparisons data are presented as mean (standard deviation) and compared using the Student-*T*-test, or as median (interquartile range, IQR) and compared using the Mann-Whitney *U*-test. Frequency distributions were analyzed by Pearson's chi-square and Fisher's exact test (two-sided). Statistical significance was defined as *p* < 0.05 for all tests.

To assess the effect of venous drainage patterns on long-term functional outcome we used multiple logistic regressions to model the probability for poor outcome while adjusting for other important clinical factors which were significant predictors of outcome in univariate analysis or considered clinically relevant a priori. The statistical AIC-criterion guided us in model evaluation. Likelihood ratio tests were used to assess statistical significance for predictors in the logistic regression. The goodness of fit of different models was evaluated based on residual plots and the Hosmer-Lemeshow test.

We performed statistical analyses using the software packages SPSS 23.0 and R 3.3.

### Approvals and consent

The study protocol was approved by the ethics committee “Ethikkommission an der Technischen Universität Dresden,” Dresden, Germany (IRB00001473; EK125042012). Written informed consent was obtained by patients or their legal representatives in accordance with the Declaration of Helsinki.

## Results

### Patients

**Figure 1:**
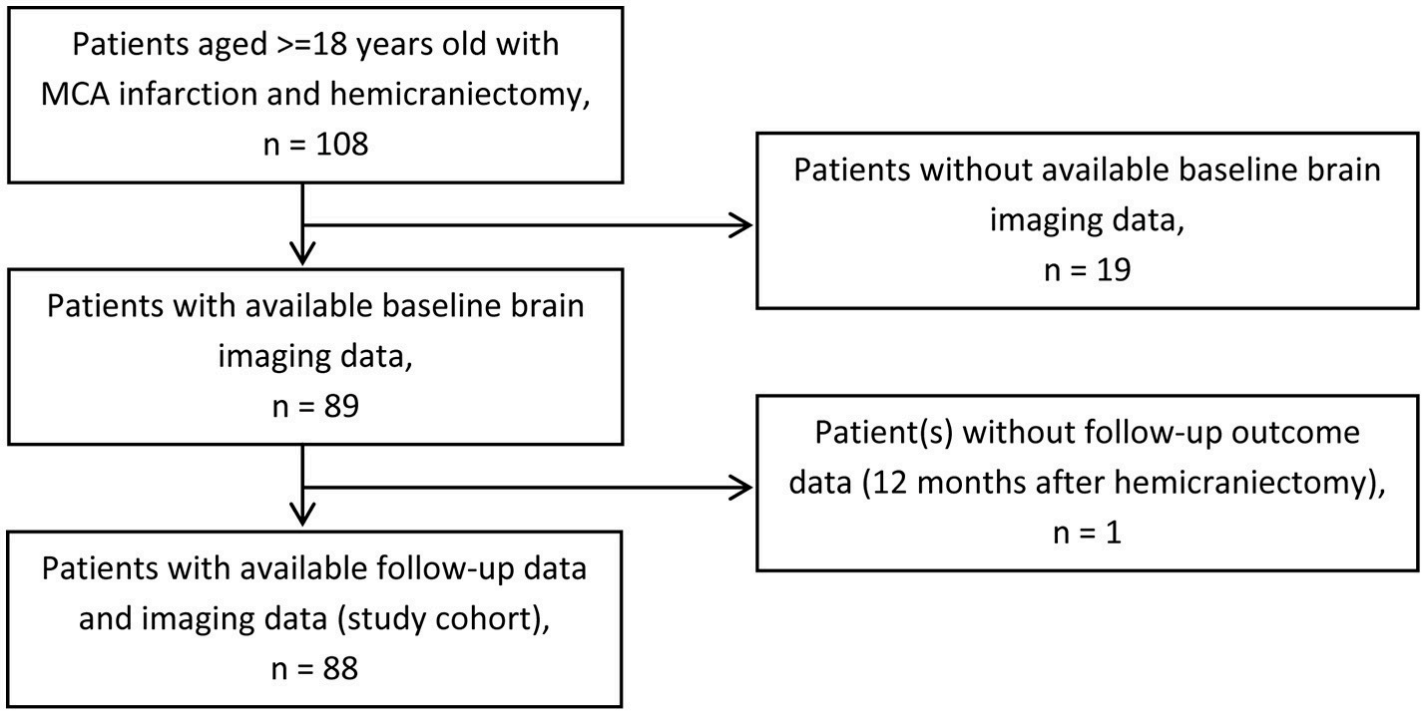
Patient selection. Flow chart of patient selection for study cohort. MCA, middle cerebral artery.

We identified 108 stroke patients with space-occupying MCA infarction who were treated with decompressive surgery at our university stroke center. Eighty eight patients with available imaging data and follow-up data were included into the study (Figure 1). Imaging data were rarely available for patients of the early study period.

Demographic and clinical characteristics, treatment course, and outcome are summarized in Table 1. Patients were predominantly male (64%), mean age was 53 years, and the dominant hemisphere was affected in 53% of patients. Median time from symptom onset to decompressive surgery was 31 h. Most patients received treatment with osmotherapy (*n* = 63) and/or hypothermia (*n* = 74).

**Table 1:** Baseline characteristics, ICU treatment and functional outcome.

		**All patients (*n* = 88)**	**Patients with mRS 0–4 at 12 months (*n* = 44)**	**Patients with mRS 5-6 at 12 months (*n* = 44)**	***p*-value**
Age (years)	mean (SD)	52.8 (8.6)	50.6 (7.8)	54.9 (8.9)	0.024
	median (IQR)	53.5 (46–59)	49 (45.25–58.25)	55.5 (47.25–60)	.
Sex (female)	*n* (%)	32 (36.4)	15 (34.1)	17 (38.6)	0.658
Hemisphere (dominant)	*n* (%)	47 (53.4)	23 (52.3)	24 (54.5)	0.831
NIHSS admission	median (IQR)	19 (15-32)	17 (14-20.75)	22.5 (17.25-32)	<0.001
Atrial fibrillation	*n* (%)	28 (31.8)	11 (25.0)	17 (38.6)	0.170
Sympton onset to DS (hours)	median (IQR)	30.5 (22–51.1)	33 (23.5–52.5)	26.0 (21–47.5)	0.167
Osmotherapy	*n* (%)	63 (71.6)	30 (68.2)	33 (75.0)	0.478
Barbiturates	*n* (%)	19 (21.6)	5 (11.4)	14 (31.8)	0.020
Hypothermia	*n* (%)	74 (84.1)	36 (81.8)	38 (51.4)	0.560
Pneumonia	*n* (%)	72 (81.8)	39 (88.6)	33 (75.0)	0.097
Sepsis	*n* (%)	18 (20.5)	7 (15.9)	11 (25.0)	0.290
Venous thrombosis	*n* (%)	8 (9.1)	3 (6.8)	5 (11.4)	0.458
Renal replacement therapy	*n* (%)	3 (3.4)	2 (4.5)	1 (2.3)	0.557
Hospital stay (days)	median (IQR)	19 (13.3–25)	20 (17–25)	16.5 (8.25–24.75)	0.038
In-hospital lethality	*n* (%)	20 (22.7)	.	.	NA
mRS score at 12 months	median (IQR)	4.5 (3–6)	.	.	NA
mRS score 0–3 at 12 months	*n* (%)	23 (26.1)	.	.	NA
mRS score 0–4 at 12 months	*n* (%)	44 (50)	.	.	NA
Lethality at 12 months	*n* (%)	30 (34.1)	.	30 (68.2)	NA

*DS, decompressive surgery; ICU, intensive care unit; mRS, modified Rankin Scale; NA, not applicable*.

Twenty of 88 patients died during intensive care unit stay (cerebral herniation, *n* = 14; medical complications, *n* = 5; hematoma after hemicraniectomy, *n* = 1). At 12 months after stroke onset, 50% of the patients had a mRS score of 0–4 and 30 (34%) patients had died.

### Baseline imaging data and venous drainage pattern

Pre-operative cerebral imaging revealed a median (IQR) ASPECT score of 0 (0–2), an additional anterior cerebral artery (ACA) infarction in 24% and an additional posterior cerebral artery (PCA) infarction in 11.4% of patients (Table 2). The median (IQR) diameter of hemicraniectomy was 121 (115–130) millimeters.

**Table 2:** Imaging of space-occupying infarction and cerebral venous drainage.

			**All patients (*n* = 88)**	**mRS 0-4 at 12 months (*n* = 44)**	**mRS 5-6 at 12 months (*n* = 44)**	***p*-value**
ASPECTS, *n* = 80		median (IQR)	0 (0-2)	1 (0-2)	0 (0-2)	0.892
Additional ACA infarction, *n* = 80		*n* (%)	21 (23.9)	7 (17.5)	14 (35)	0.075
Additional PCA infarction, *n* = 80		*n* (%)	10 (11.4)	3 (6.8)	7 (15.9)	0.313
Additional ACA and/or PCA infarction, *n* = 80		*n* (%)	29 (33)	10 (22.7)	19 (43.2)	0.043
Decompressive surgery, diameter, *n* = 86	mm	median (IQR)	121 (115–130)	122 (116–131)	119 (112–130)	0.199
**VENOUS DRAINAGE IPSILATERAL TO MCA INFARCTION**						
Ipsilateral internal jugular vein	Occlusion	*n* (%)	0	0	0	0.799
	Hypoplasia	*n* (%)	16 (18.2)	7 (15.9)	9 (20.5)	
	Normal	*n* (%)	52 (59.1)	26 (59.1)	26 (59.1)	
	Not assessable	*n* (%)	20 (22.7)	11 (25.0)	9 (20.5)	
Ipsilateral transverse sinus	Occlusion	*n* (%)	1 (1.1)	0	1 (2.3)	0.275
	Hypoplasia	*n* (%)	25 (28.4)	10 (22.7)	15 (34.1)	
	Normal	*n* (%)	62 (70.5)	34 (77.3)	28 (63.6)	
Ipslateral combined	Occlusion/hypoplasia	*n* (%)	27 (30.7)	11 (25.0)	16 (36.4)	0.248
**VENOUS DRAINAGE CONTRALATERAL TO MCA INFARCTION**						
Contralateral internal jugular vein	Occlusion	*n* (%)	1 (1.1)	0	1 (2.3)	0.719
	Hypoplasia	*n* (%)	15 (17.0)	8 (18.2)	7 (15.9)	
	Normal	*n* (%)	52 (59.1)	25 (56.8)	27 (61.4)	
	Not assessable	*n* (%)	20 (22.7)	11 (25.0)	9 (20.5)	
Contralateral transverse sinus	Occlusion	*n* (%)	1 (1.1)	0	1 (2.3)	0.576
	Hypoplasia	*n* (%)	25 (28.4)	12 (27.3)	13 (29.5)	
	Normal	*n* (%)	62 (70.5)	32 (72.7)	30 (68.2)	
Contralateral combined	Occlusion/hypoplasia	*n* (%)	26 (29.5)	12 (27.3)	14 (31.8)	0.640
**ANY OCCLUSION OR HYPOPLASIA OF TRANSVERSE SINUSES OR INTERNAL JUGULAR VEINS**						
	Occlusion/hypoplasia	*n* (%)	50 (56.8)	23 (52.3)	27 (61.4)	0.389
Imaging modality						
	CTA	*n* (%)	35 (39.8)			
	MRI (without contrast)	*n* (%)	5 (5.7)			
	Angiography (DSA)	*n* (%)	4 (4.6)			
	NCCT	*n* (%)	19 (21.6)			
	Multiple modalities (including CTA/DSA)	*n* (%)	20 (22.7)			
	Multiple modalities (without CTA/DSA)	*n* (%)	5 (5.7)			

*ACA, anterior cerebral artery; PCA, posterior cerebral artery; DS, decompressive surgery; CTA, computed tomography angiography; MRI, magnetic resonance imaging; DSA, digital subtraction angiography; NCCT, non-contrast computed tomography; occl, occlusion*.

**Figure 2:**
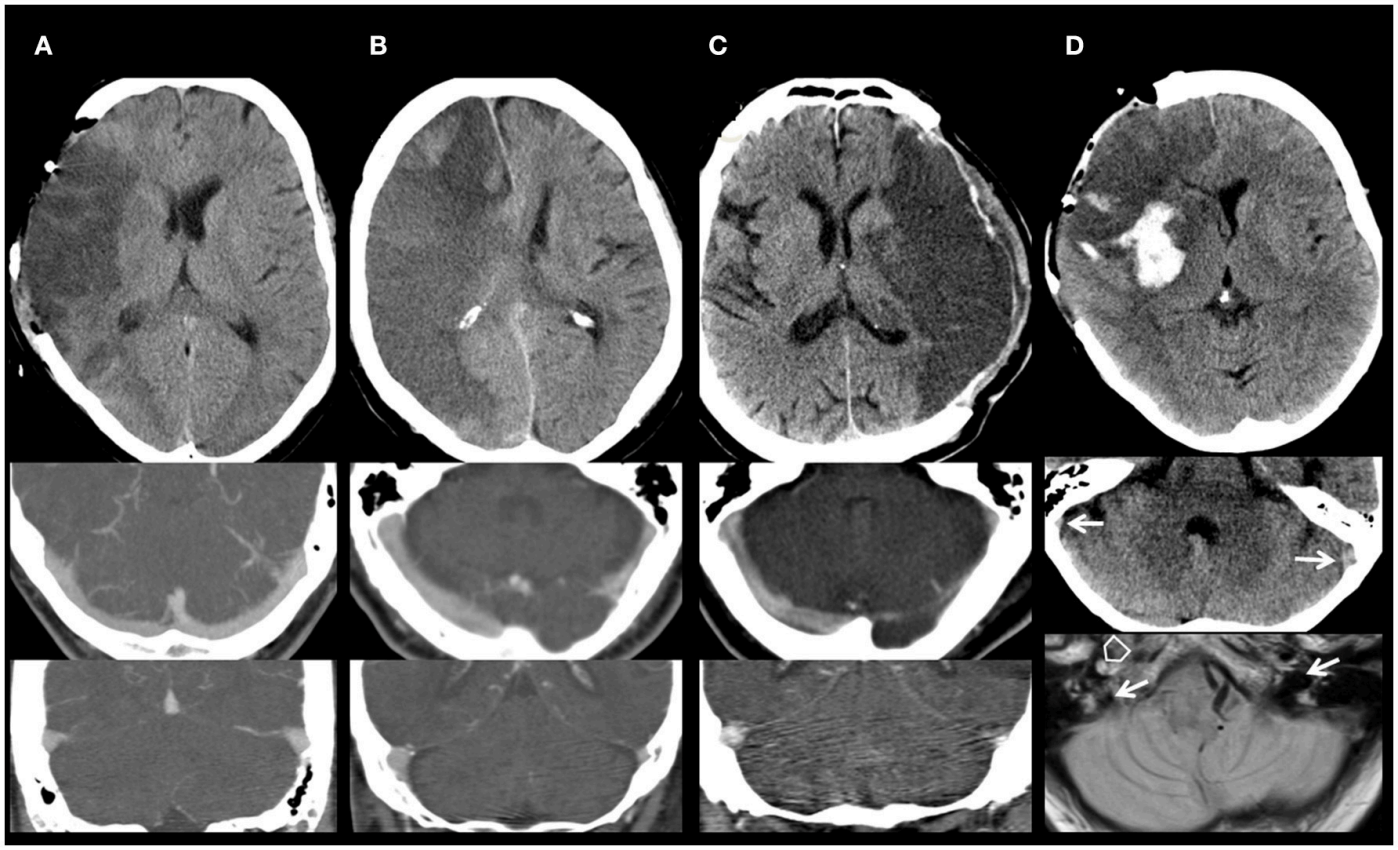
Imaging of cerebral venous drainage. **(A)** Normal appearing venous drainage. Non-contrast computed tomography (NCCT) of a 60-year-old man with a large right middle cerebral artery (MCA) infarction after decompressive surgery. Symmetric venous drainage by both ipsilateral and contralateral transverse sinus and internal jugular veins (CTA). **(B)** Abnormal contralateral venous drainage. NCCT of a 66-year-old woman with a space-occupying right MCA and anterior cerebral artery infarction. Hypoplasia of the contralateral transverse sinus and internal jugular vein (CTA). **(C)** Abnormal ipsilateral venous drainage. NCCT of a 60-year-old man with a large left MCA infarction who underwent decompressive surgery. Hypoplasia of the ipsilateral transverse sinus and internal jugular vein (CTA). **(D)** Abnormal ipsilateral venous drainage. Imaging of a 62-year-old woman with a large right MCA infarction, after decompressive surgery (NCCT; Upper). Hypoplasia of the ipsilateral transverse sinus and internal jugular vein. NCCT (Middle): Asymmetry of the sigmoid sinus, hypoplastic right, dominant left (arrows). MRI (proton density; Lower): basal scan close to the foramen magnum. Open arrow: occluded right internal carotid artery (missing flow void). Filled arrows: dominant left internal jugular vein (IJV), hypoplastic right IJV.

For the analysis of the venous drainage pattern we used cerebral imaging including CTA or DSA in 67% of the patients and NCCT or MRI in the remaining patients (Table 2, Figure 2). Image analysis of CVD patterns revealed a hypoplasia or occlusion in one or more of the evaluated venous segments in 50 of 88 patients (57%) and in 84 of 312 (30%) venous segments evaluated (Table 2). Hypoplasia or occlusion ipsilateral to the side of the infarction was found for the transverse sinus in 26 of 88 (30%) patients and for the internal jugular vein in 16 of 88 (18%) patients. A contralateral hypoplasia or occlusion was found for the transverse sinus in 30% and for the internal jugular vein in 18% of patients. Normal appearing transverse sinus and internal jugular vein were present in 59 and 71% of patients on the ipsilateral and contralateral side to the infarction, respectively.

### Group comparison of patients with poor outcome at 12 months (mRS 5 and 6) and patients with mRS 0–4

Apart from higher baseline NIHSS scores and lower age, demographic factors and treatment characteristics were comparable in patients with good (mRS score 0–4) and poor (mRS score 5–6) functional outcome (Table 1). The extent of baseline MCA ischemia as quantified with ASPECTS was not different between both groups, but an additional infarction of the ACA and/or PCA territory was more frequently present in patients with poor outcome (Table 2).

The ipsilateral transverse sinus tended to be more often occluded or hypoplastic in patients with poor outcome compared to patients with good functional outcome [15/44(34%) vs. 10/44 (23%), respectively; *p* = 0.275]. The rate of venous occlusion or hypoplasia of the ipsilateral internal jugular vein was comparable in patients who had a poor outcome compared to patients with a favorable outcome [9/44(21%) vs. 7/44(16%), respectively; *p* = 0.799). Occlusion or hypoplasia of the contralateral transverse sinus [12/44(27%) vs. 14/44(32%), respectively; *p* = 0.576], or the contralateral internal jugular vein [8/44 (18%) vs. 8/44 (18%)] were distributed similarly between patients with poor outcome compared to patients with good functional outcome.

### Association of abnormal cerebral venous drainage with functional outcome at 12 months

In univariate analyses, abnormal CVD patterns showed non-significant associations with poor functional outcome at 12 months: ipsilateral abnormal CVD (i.e., hypoplasia or occlusion of internal jugular vein or transverse sinus), Odds ratio [OR] 1.98 (95%CI 0.75–5.40); contralateral abnormal CVD, OR 1.56 (95%CI 0.59–4.24); any abnormal CVD (ipsilateral and/or contralateral), OR 1.6 (95%CI 0.68–3.79). When any impaired CVD was defined as numeric variable (0, 1, or 2 venous vessels with impaired drainage), univariate analysis showed an increased probability for poor outcome for patients with higher numbers of venous vessels affected (OR 1.76, 95%CI 0.8–4.0), again not statistically significant.

Of other factors tested as potential predictors for poor functional outcome, only higher NIHSS score on admission (*p* = 0.004), higher patient age (*p* < 0.017) and additional infarction of ACA and/or PCA territory (*p* = 0.039) were significantly associated with poor outcome. The type of recanalization therapy, occurrence of pneumonia, and time to decompressive surgery were not significantly associated with poor outcome.

We combined the clinically relevant factors (1) higher patient age, (2) higher NIHSS score, (3) additional cerebral infarction (ACA and/or PCA territory), (4) pneumonia, and (5) ipsilateral abnormal CVD into a multiple logistic regression model for prediction of poor outcome. During model simplification guided by the statistical AIC-criterion we dropped the factor pneumonia (Table 3). Higher patient age (OR 1.07, 95%CI 1.01–1.14) and higher baseline NIHSS score (OR 3.42, 95%CI 1.31–9.40) were independent predictors of poor outcome according to this model, but not ipsilateral abnormal CVD (OR 1.31, 95%CI 0.47–3.67; *p* = 0.602).

**Table 3:** Multiple logistic regression analysis for prediction of poor functional outcome (mRS scores 5–6).

**Predictor**	**OR**	**95-% CI**	***p*-value**
Age	1.07	1.01–1.14	0.013
NIHSS	3.42	1.31–9.40	0.012
Additional ACA/PCA infarction	2.24	0.83–6.31	0.111
Ipsilateral abnormal venous drainage	1.31	0.47–3.67	0.602

*ACA, anterior cerebral artery; PCA, posterior cerebral artery*.

Testing two other logistic regression models, substituting *ipsilateral* abnormal drainage with *contralateral* and *any* abnormal drainage, the model including the factor *any* abnormal venous drainage as numeric variable showed the best AIC value of all three models. A higher number of venous vessels with abnormal drainage was associated with poor functional outcome (OR 2.01, 95%CI 0.80–5.39), but this effect was again not statistically significant. Contralateral abnormal CVD was also not an independent predictor of poor functional outcome at 12 months after hemicraniectomy.

Based on the observed proportions of poor functional outcome in patients with ipsilateral abnormal CVD and in patients with normal ipsilateral drainage in our cohort, in order to achieve a power of 80% for a 2-sided Fisher's exact test with a significance level of α = 0.05, a sample size of 456 patients would be needed.

## Discussion

The primary hypothesis of our study was that an abnormal cerebral venous drainage ipsilateral to the side of malignant MCA infarction in patients who underwent decompressive surgery would result in reduced venous drainage and consecutive venous congestion, more severe edema progression, and worse functional outcome compared to patients with normal ipsilateral venous drainage. However, in our study evaluating the CVD pattern in 88 patients with malignant MCA infarction who underwent hemicraniectomy, ipsilaterally abnormal venous drainage was not significantly associated with long-term functional outcome. Moreover, also contralaterally abnormal or any abnormal CVD were not predictive for poor functional outcome in this cohort.

Baseline characteristics of patients included in our study were comparable to characteristics of patients with space-occupying MCA infarction in previous randomized hemicraniectomy trials (6, 7). Functional outcome was not directly comparable with earlier hemicraniectomy trials due to different inclusion criteria including patient age. We found an occlusion or hypoplasia of the transverse sinus in 30% and of the internal jugular vein in 18% of patients. This is in line with earlier reports regarding human cerebral venous drainage (14–16).

The physiological role of CVD and its alterations in stroke patients have been studied rarely. After ischemic stroke by large artery occlusion the adjacent draining veins collapse due to reduced intra-vascular pressure and increased extra-vascular pressure. The reduced venous outflow might cause a redistribution of blood from the infarct core to the penumbra by collateral veins, a concept called cerebral blood steel phenomenon (17). The venous collateral status might therefore influence the development of ischemic brain edema. However, it is unclear whether patients with hypoplastic or aplastic venous sinuses have compensatory venous collaterals more frequently.

Some studies have focused on CVD and its impact on the prognosis of ischemic stroke. Using mono-phase CT angiography in stroke patients, impaired filling of deep and cortical veins in territories corresponding to the infarction was associated with poor functional outcome (18–20). Furthermore, asymmetric venous drainage in stroke patients was associated with larger areas of hypoperfusion and with worse clinical outcome (21). In a case series including 14 stroke patients, abnormal ipsilateral cranial venous drainage was present in 4 of 5 patients who developed fatal brain edema after MCA infarction (11). Although we could not detect a significant association between occlusion or hypoplasia of large venous segments and worse long-term functional outcome in patients with large MCA stroke, venous aplasia or hypoplasia were more frequently observed in patients with poor functional outcome. This was observed for all three composite variables (ipsilateral, contralateral, any abnormal cerebral venous drainage).

Similarly to previous studies, higher patient age and baseline stroke severity were independent predictors for poor functional outcome in our cohort (9). A score for stroke patients with MCA occlusion has been introduced recently to assess the probability of malignant brain edema (22). This Malignant Brain Edema Score incorporates (1) the baseline NIHSS Score, (2) the ASPECT Score, (3) the Clot Burden Score, grading the extent of arterial clotting, (23) (4) ipsilateral arterial collateral status, and (5) the large vessel recanalization status. Based on our study results, an incorporation of CVD state in prediction scores for long-term functional outcome after severe MCA stroke seems currently not justified.

About one-third of patients of the cohort had an additional infarction of the ACA/PCA territory, reflecting clinical practice to select patients for hemicraniectomy at our center during the study period. In previous studies, higher MCA infarction volume and occurrence of additional ACA/PCA infarction were shown to be independent predictors for poor outcome in patients with MCA infarction and hemicraniectomy (24, 25). Additional ACA/PCA infarction was more frequently observed in patients with poor outcome in our cohort, although not independently predictive for outcome, which is attributable in part to the number of patients included in our cohort. However, MCA infarction volume and/or additional infarctions need to be taken into account if hemicraniectomy is considered in patients with MCA infarction (26).

Our study has strengths and limitations. We report one of the largest case series of surgically treated patients with space-occupying MCA stroke who were evaluated for cerebral venous drainage. Furthermore, the selected stroke patients were routinely treated according to an institutional protocol during the observational period. The image analyses for the study were performed in a three reader consensus mode, limiting misinterpretation of imaging data.

Limitations of our study arise from the retrospective, single-center study design. Patients with MCA infarction were selected for the study cohort from a basic hemicraniectomy cohort by availability of imaging data, which was limited for the early time period considered, so selection bias may have influenced our results. The evaluation of functional outcome was performed retrospectively. However, we applied a definition of poor functional outcome as modified Rankin Scale scores of 5 or 6 which are both conditions that can reliably retrieved retrospectively. Another important limitation of our analysis is the size of our cohort. The regression analyses revealed associations of different patterns of abnormal CVD with poor functional outcome, but each not reaching statistical significance. Although the cohort was relatively large (*n* = 88), the number of patients included may have prevented the detection of statistically significant associations of abnormal CVD with outcome measures due to the lack of statistical power. Further studies with larger sample sizes should be undertaken to further evaluate the effect of CVD in patients with malignant MCA infarction.

Different imaging modalities with varying power for the detection of CVD patterns were used in our analyses. CTA was primarily used to detect arterial occlusions and not to evaluate venous drainage pattern, therefore interpretation of venous drainage using arterial-phase CTA is limited. As we used static evaluations and not a sequential or dynamic evaluation of CVD, the results should be interpreted with caution. The term “abnormal” drainage, used in our study for patients with aplastic, hypoplastic or occluded venous vessels, is based on anatomical and not on functional data, respectively. The combined dynamic and static contrast-enhanced MR venography has been previously been shown to accurately identify impaired cerebral venous flow and venous thrombosis (27, 28).

Furthermore, we used cerebral infarction patterns as imaging surrogate for impaired cerebral arterial influx, as angiographic data were available for only 67% of patients. Individual cerebral arterial flow patterns and intra-individual differences between both hemispheres might have contributed to edema progress and functional outcome in our cohort.

In conclusion, stroke patients with space-occupying MCA infarction who underwent decompressive surgery, an impaired cerebral venous drainage was observed more frequently in patients with poor functional outcome, but no significant associations of drainage patterns with clinical outcome measures were found. Prospective studies with larger sample sizes and using dynamic imaging modalities could further determine the role of venous drainage in patients with space-occupying MCA infarction.

## Data availability statement

The dataset used for analyses reported in this manuscript is not publicly available because consent statements of study patients and local data protection guidelines restrict its use to investigators of the Technische Universität Dresden, Germany, and their affiliates.

## Author contributions

VP designed the study, acquired and interpreted data, and revised the manuscript for content. JG acquired and interpreted data and revised the manuscript for content. PK acquired and interpreted data and revised the manuscript for content. MK interpreted data, performed statistical analyses, and revised the manuscript for content. HR interpreted data and revised the manuscript for content. HS has designed the study, acquired and interpreted data, performed statistical analyses, and drafted the manuscript. All authors read and approved the final manuscript.

### Conflict of interest statement

The authors declare that the research was conducted in the absence of any commercial or financial relationships that could be construed as a potential conflict of interest.
